# Oncogenic Fli-1 is a potential prognostic marker for the progression of epithelial ovarian cancer

**DOI:** 10.1186/1471-2407-14-424

**Published:** 2014-06-12

**Authors:** Wei Song, Lingyun Hu, Wei Li, Guanjun Wang, Yan Li, Lei Yan, Ailing Li, Jiuwei Cui

**Affiliations:** 1 Cancer center, the First Hospital of Jilin University, 71 Xinmin Street, Changchun 130021, China; 2 Obstetrics and Gynecology, the General Hospital of Chinese People’s Liberation Army, Beijing, China; 3 Institute of Basic Medical Sciences, National Center of Biomedical Analysis, 27 Tai-Ping Road, Beijing 100850, China

**Keywords:** Epithelial ovarian cancer (EOC), Fli-1, Tumor stage, Overall survival

## Abstract

**Background:**

Ovarian cancer is the most lethal gynecologic malignancy, but its etiology remains poorly understood. This study investigated the role of Fli-1 in ovarian carcinogenesis and disease survival.

**Methods:**

Fli-1 protein expression was evaluated by immunohistochemistry in 104 primary epithelial ovarian cancer (EOC) patients with known follow-up data and 20 controls. Correlation between Fli-1 expression and clinical characteristics was evaluated with the logistic regression. Kaplan Meier analysis was used to assess the impact of Fli-1 expression on overall survival (OS) and disease-free survival (DFS). Cell proliferation and migration assay were used to explore the function of Fli-1 in ovarian cancer cells.

**Results:**

Fli-1 was expressed in 74% cases and up-regulated in EOC tissues compared with normal control tissues (*p*< 0.05). The high expression of Fli-1 was significantly associated with advanced tumor stage, positive lymph nodal involvement, and poor OS and DFS (*p*< 0.05). Further analysis showed Fli-1 is an independent prognostic factor for OS and DFS. Down-regulation of Fli-1 inhibited cell proliferation but did not affect cell migration in SKOV3 cells.

**Conclusions:**

This study revealed that Fli-1 played an essential role in the development and progression of ovarian cancers. Its overexpression is intimately related to malignant phenotypes and poor clinical outcome, suggesting that Fli-1 is a potential prognostic marker and therapeutic molecular target in ovarian cancer.

## Background

Ovarian cancer is the leading cause of death from gynecologic malignancy in developed countries and the second leading cause in developing countries [1,2]. Epithelial ovarian cancer (EOC) accounts for 90% of ovarian cancers; however, its aetiology remains unknown. The origin and pathogenesis of EOC have been investigated but still poorly understood. Over the past decades, prognosis for patients with EOC has improved little,with 70–80% of the cases having a recurrence of the cancer and ultimately succumbing to the disease [3]. There are a number of genetic and epigenetic changes that lead to transformation of ovarian epithelial cells into tumor cells [4]. Recognizing the importance of molecular mechanism, it is urgent to identify key molecular regulators in tumorigenesis to improve the prognosis assessment and treatment of EOC patients.

Friend leukemia virus integration 1 (Fli-1), a member of the ETS transcription factor family, is the target of insertional activation by Friend murine leukemia virus (F-MuLV) and is preferentially expressed in vascular endothelial cells and hematopoietic tissues [5,6]. Transcription factors of the ETS family regulate the expression of oncogenes, tumor suppressor genes, and some related genes of the vessel’s formation, invasion and metastasis, and often correlate with poor survival in some types of cancers [7-10]. Fli-1 plays a critical role in normal development, hematopoiesis and oncogenesis by functioning as both transcriptional activator and repressor [11-15]. Knocking-down Fli-1 expression in erythroleukemic cells leads to a marked growth inhibition and cell death, demonstrating a possible therapeutic approach to induce tumor suppression [16-19].

It has also been shown that Fli-1 maintains several malignant phenotypes by inhibiting Rb, GATA-1, SHIP-1 and targeting Bcl-2 [20-22]. Anti-Fli-1 compounds had been discovered and demonstrated strong anti-leukemic activity in a mouse erythroleukemia model that overexpresses Fli-1, making it possible for targeting Fli-1 as a potent treatment strategy [23].

However, the role of Fli-1 in EOC remains unknown. Here, we analyzed Fli-1 expression in EOC patients and studied its function in an ovary cell line.

## Methods

### Patients and samples

Formalin-fixed paraffin-embedded tissues of ovary and fallopian tube from primary ovarian cancer patients and control group, such as uterine prolapse, uterine fibroid were obtained from the First Hospital of Jilin University and the General Hospital of Chinese People’s Liberation Army between 2005 and June 2009. The specimens included 104 Ovarian Serous Carcinoma, 10 fallopian tube and 10 normal ovaries. Primitive neuroectodermal tumor (PNET) was chosen as the positive controls at the same time. Clinical stage, histological grade and follow-up data were available for the majority of these patients. The histological subtypes and disease stages of the tumors were classified according to International Federation of Gynecology and Obstetrics (FIGO) criteria. Approval for the study was obtained from the Research Ethics Board of the First Hospital of Jilin University and the General Hospital of Chinese People’s Liberation Army. The study participants gave their written informed consent. The clinical characteristics of all patients and the control group were shown in Table 1. The patients were followed up for survival analysis.

**Table 1:** Clinicopathologic characteristics of EOC patients

**Variable**	**Total number %**	
Age median (range)	52 (22–73)	
< 50	43	41
≥ 50	61	59
FIGO stage		
I	20	19
II	16	16
III	50	48
IV	18	17
Histological grade		
G1	10	10
G2	14	13
G3	80	77
Lymph nodal involvement		
Positive	40	38
Negative	64	62
Residual tumor size		
0 mm	76	75
0–10 mm	28	25
CA125 serum level median (range)	263 (19–8410)	
≤ 35 U/ml	7	7
> 35 U/ml	97	93
ER expression		
Positive	58	56
Negative	46	44
PR expression		
Positive	47	45
Negative	57	55
Her1 expression		
Positive	37	36
Negative	67	64
Her2 expression		
Positive	51	49
Negative	53	51
P53 expression		
Positive	73	70
Negative	31	30

### Immunohistochemistry

Tissue slides were de-paraffinized with xylene and rehydrated through a gradual decline of alcohol (100–80%), and then incubated in 3% hydrogen peroxide for 15 minutes to block endogenous peroxidase activity. Antigen retrieval was carried out by immersing the slides in 10 mM sodium citrate buffer (pH 6.0) and maintained at a sub-boiling temperature for 15 minutes. The slides were rinsed in phosphate-buffered saline and incubated with 10% normal goat serum to block non-specific staining for 30 minutes at 37°C. Primary anti-Fli-1 polyclonal antibodies (Neomarker) were diluted in 1:100, and incubated with the sections at 4°C overnight. After washing with PBS, the secondary antibodies (biotinylated goat anti-rabbit immunoglobulin) and streptavidin peroxidase complex reagent were applied. Subsequently, the visualization signal was processed according to the Polink-2 HRP DAB Detection kit. Finally, the slides were counterstained with hematoxylin for 15 min and dehydrated in ascending concentrations of alcohol (80–100%). After xylol treatment, slides were covered.

Two investigators evaluated each stained section independently without knowing any clinical information. The proportions of positive cells were ranged from 10 to 100%, while the intensity of staining was scored as 0 (negative), 1 (weak), 2 (moderate), and 3 (intense) in the most strongly stained tumor area. The immunoreactivity score for each case was taken as percentage of positive cells multiplied by the intensity of staining.

### RNA interference and transfection

Fli-1-specific siRNAs (No. 1 and No. 2) were from Invitrogen. The target sequences were 5′-GGGAAAGUUCACUGUUGGCCUAUAA-3′ and 5′-AGGAGUGGAUCAAUCAGCCAGU-GAG-3′, respectively. The target sequence of control siRNA against photinus pyralis luciferase gene (Invitrogen, CA) was 5′-GGAUUUCGAGUCGUCUUAAUGUAUA-3′. RNAiMAX transfection reagent was used for transient transfection following manufacturer’s protocol (Invitrogen, CA).

### Cell proliferation assay

SKOV3 cells were maintained in DMEM containing 1% penicillin and streptomycin, supplemented with 10% fetal bovine serum (FBS), then incubated overnight at 37°C, 5% CO_2_ with density 10% per well. The number of cell proliferation was measured by Trypan-blue exclusion assay from day 1 to day 4.

### Cell migration and invasion assay

Cell migration and invasion assays were carried out using Transwell (Corning Costar Corp, MA) membrane filter inserted in 24-well tissue culture plates (6.5-mm diameter, 8-μm pore size). For migration assay, cells (4 × 10^4^) suspended in serum-free medium were seeded on the upper chamber of transwell filters. Serum-containing medium was added to the lower chamber and incubated for 16 h at 37°C. Nonmigrating cells were removed by wiping the upper side of the filter, and the remaining cells on the lower surface of the filter were fixed with 4% formaldehyde, stained with crystal purple and counted under a microscopy. Each determination represents the average of three individual experiments.

### Immunoblotting and antibodies

Cells were lysed with radioimmunoprecipitation assay buffer (1% Nonidet P-40, 50 mM Tris–HCl, pH 7.4, 150 mM NaCl, 1% sodium deoxycholate, 0.1% SDS, plus protease inhibitor cocktail and 1 mM phenylmethylsulfonyl fluoride). Proteins were separated by SDS-PAGE and analyzed by Western blotting. Antibodies to Fli-1 and β-actin were obtained from Santa Cruz Biotechnology (Santa Cruz Biotechnology, CA, USA).

### Cytoplasmic and nuclear fractionation

Cells were harvested by trypsin-EDTA, collected by centrifugation and washed two times in ice-cold PBS. Pellets were lysed in buffer A containing 10 mM HEPES, pH 7.9, 10 mM KCl, 0.1 mM EDTA, 0.1 mM EDTA, 1 mM PMSF, 1 mM DTT, 1 mM Na3VO4 supplemented with protease inhibitors and incubated for 15 min on ice. Thereafter, NP-40 was added at a final concentration of 10% and lysates were oscillated. Nuclei were pelleted by centrifugation at 1000 g for 1 min at 4°C and supernatant containing cytoplasmic proteins (C). The nucleic pellets were lysed in buffer B containing 20 mM HEPES, pH 7.9, 0.4 M NaCl, 1 mM EDTA, 1 mM EGTA, 1 mM PMSF, 1 mM Na3VO4, 1 mM DTT, supplemented with protease inhibitors by repeated freezing and oscillating. Supernatants containing soluble nucleic proteins (N) were collected by centrifugation at 12000 g for 10 min.

### Statistical analysis

Statistical analysis was performed by using univariate (nonparametric rank sum test) and multivariate (logistic regression) analysis to evaluate the relationship between gene expression and clinicopathological parameters, including age, FIGO stage, histological grade, lymph nodal involvement, residual tumor size, CA125, ER expression, PR expression and P53 expression. Disease Free Survival (DFS) and Overall Survival (OS) were calculated by using the Kaplan-Meier method, and the differences were assessed by using the log-rank test. Comparison was made of groups with high Fli-1 expression (score > median score) and low Fli-1 expression (score ≤ median score). The nonparametric rank sum test was used to determine the significance of the difference in the distribution of gene expression in cancer, borderline and normal samples. These analyses were performed by SPSS 13.0 Statistical Software. *P* ≤ 0.05 was considered as statistically significant.

## Result

### Oncogenic Fli-1 is up-regulated in ovarian cancer tissues

**Figure 1:**
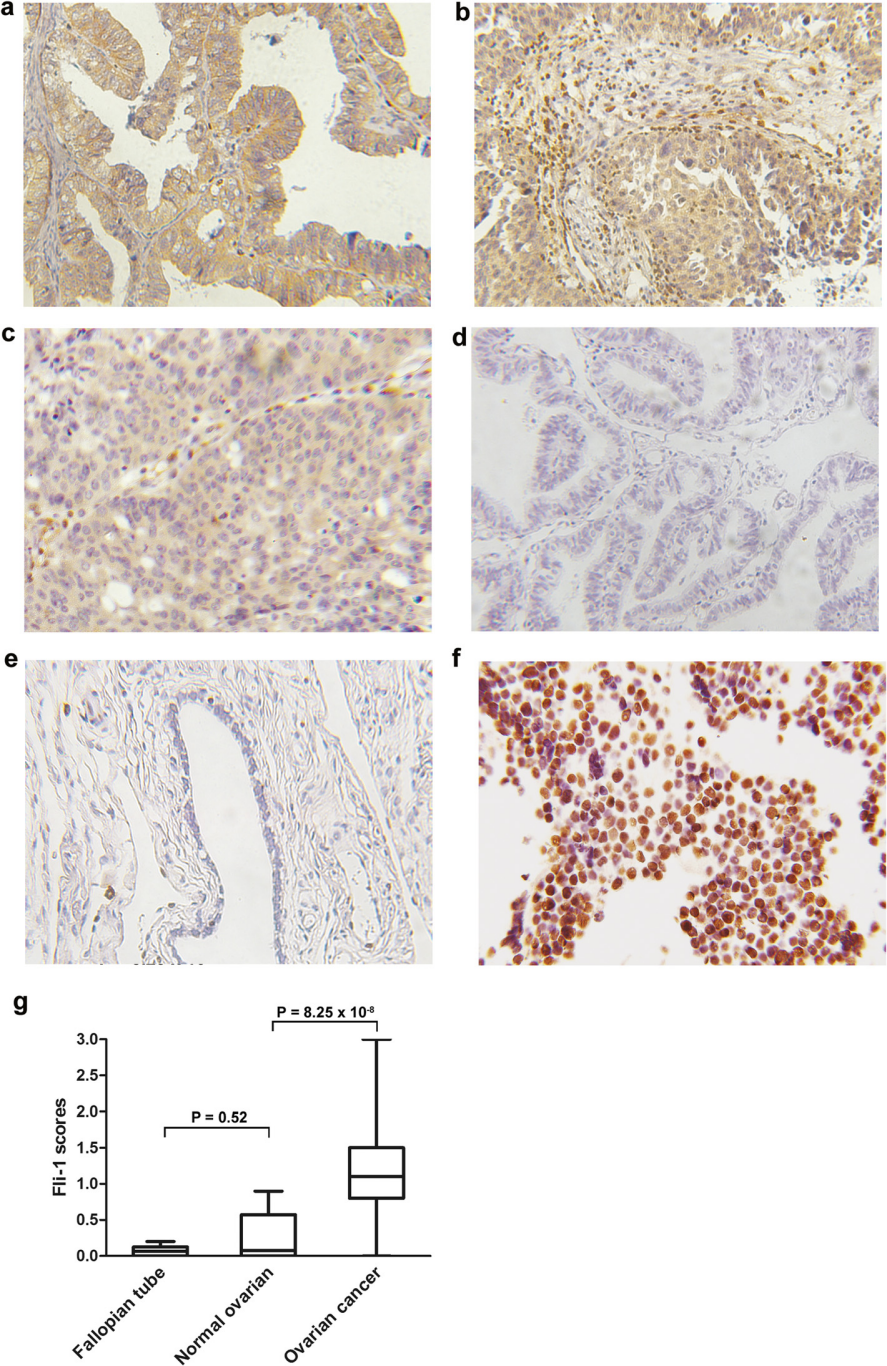
**Fli-1 is highly expressed in ovarian cancer. (a, b, c)**. Representation of images from immunohistochemical stains Fli-1 in tumors from three cases of ovarian cancer. **(d)** Expression of Fli-1 in fallopian tube was negative. **(e)** Expression of Fli-1 in normal ovarian tissue was negative. Original magnification × 200. **(f)** Fli-1 were positive in nuclear in PNET tissues. PNET: Primitive neuroectodermal tumor. **(g)** Fli-1 expression scores are shown as box plots, in ovarian cancer, fallopian tube and normal ovarian tissue. with the horizontal lines representing the median; the bottom and top of the boxes representing the 25^th^ and 75^th^ percentiles, respectively; and the vertical bars representing the range of data.

Immunohistochemical staining revealed that Fli-1 was generally expressed in the cytoplasm of ovarian cancer cells with various intensities (Figure 1). In primitive neuroectodermal tumor (PNET), Fli-1 was positive in nuclear (Figure 1f). Of the 104 EOC specimens examined in this study, Fli-1 was positive in 77 (74%) cases. The scores of intensity were also analyzed. Eight (7.7%) cases lacked Fli-1 expression; 19 (18.3%) demonstrated weak expression of Fli-1; 60 (57.7%) demonstrated moderate expression of Fli-1 and 17 (16.34%) demonstrated a strong signal. Compared to EOC tissues, Fli-1 expression was either negative or expressed at negligible amount in normal ovaries and fallopian tube tissues. The expression levels of Fli-1 was significantly up-regulated in EOC tissues compared with normal ovarian (*p* = 4.56 × 10^−5^) and fallopian tube tissues (*p* = 8.25 × 10^−6^).

### Fli-1 expression is associated with clinicopathological characteristics of patients with EOC

**Figure 2:**
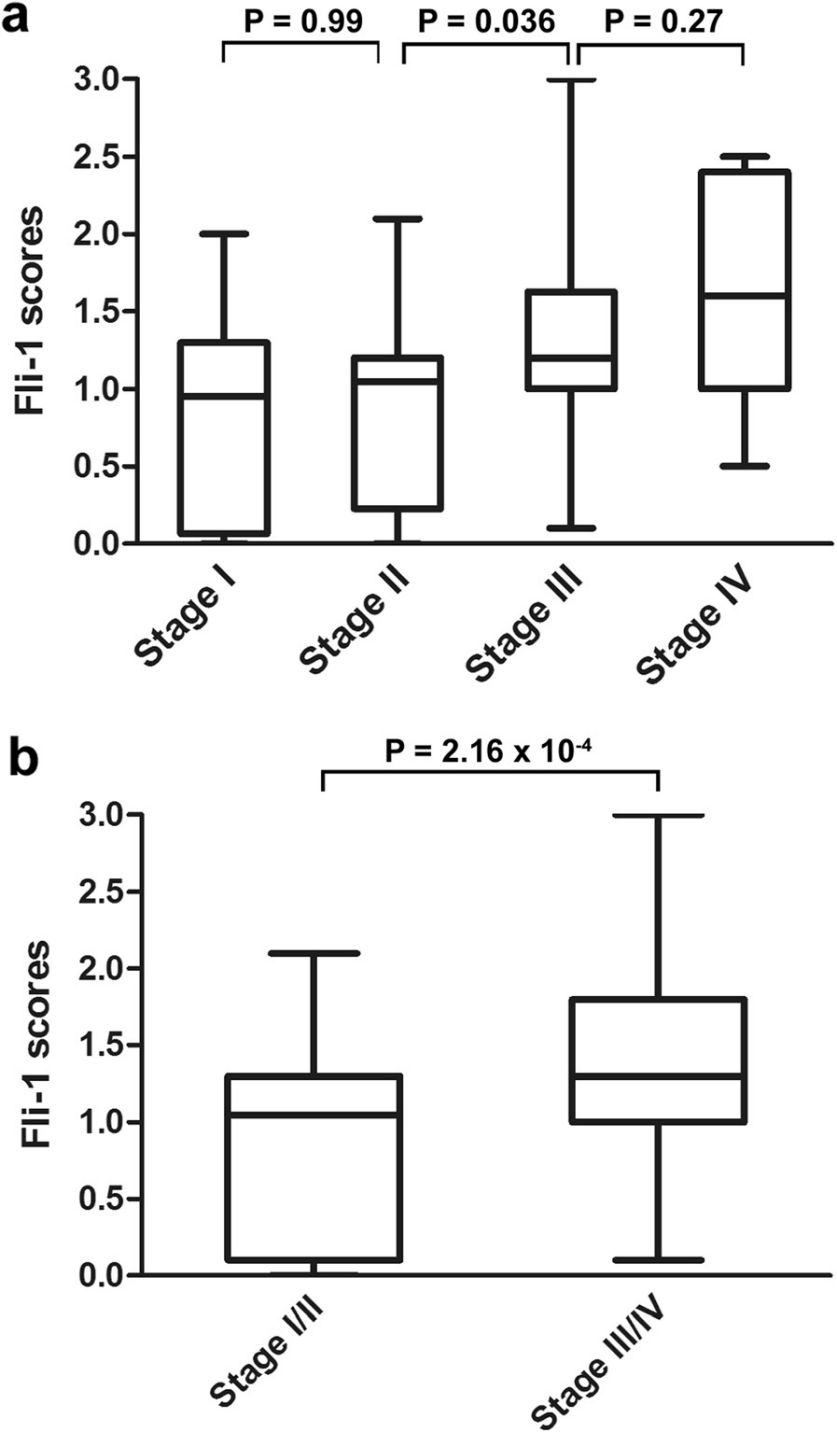
**Inverse correlation between Fli-1 expression and tumor stage. (a)** Box plot of Fli-1 expression in tumors with different stage. **(b)** Box plot of Fli-1 expression in low stage and advanced stage.

The expression rates of Fli-1 were 50.0%, 62.4%, 84% and 83.3% in stage I, II, III and IV, respectively. There was no difference in the Fli-1 scores of expression level between stageIand stageII, and between stage III and stage IV. However, there was a significant difference between stageII and stage III (*p* = 0.036, Figure 2a). Furthermore, the EOC tissues with advanced stage (III ~ IV) showed high Fli-1 expression more frequently than those with early stage (I ~ II) (*p* = 0.000216, Figure 2b).

The relationship between Fli-1 expression and clinicopathologic parameters analyzed by univariate and multivariate analysis was illustrated in Table 2. The expression of Fli-1 was significantly increased in the group of FIGO stage III and IV, lymph nodal involvement, and CA125 serum level > 35 U/ml (p < 0.05). However, Fli-1 expression was not correlated with age, histological grade, residual tumor size, and the expression of ER, PR, Her1, Her2 and P53 (*p* > 0.05).

**Table 2:** Association between Fli-1 expression and clinicopathological parameters

**Parameter**	**p-value (uni)**	**p-value (multi)**	**95% CI**	**OR**
Age at diagnosis	0.848	0.485	0.70-2.13	1.22
< 50 vs ≥ 50				
FIGO stage	< 0.010	< 0.010	0.12-0.52	0.25
≤II vs > II				
Histological grade	0.309	0.153	0.18-1.31	0.48
G1 vs G2&G3				
Lymph nodal involvement	0.015	< 0.010	0.23-0.80	0.43
Positive vs negative				
CA125 serum level	0.006	0.013	0.04-0.68	0.16
≤ 35 U/ml vs > 35 U/ml				
Residual tumor size	0.341	0.421	0.41-1.45	0.77
0 mm vs 0-10 mm				
ER expression	0.575	0.425	0.46-1.39	0.80
Positive vs negative				
PR expression	0.875	0.872	0.60-1.82	1.05
Positive vs negative				
Her1 expression	0.228	0.230	0.80-2.58	1.43
Positive vs negative				
Her2 expression	0.747	0.518	0.69-2.08	1.20
Positive vs negative				
P53 expression	0.586	0.836	0.59-1.94	1.07
Positive vs negative				

### Fli-1 is a potential prognostic biomarker for ovarian cancer survival

**Figure 3:**
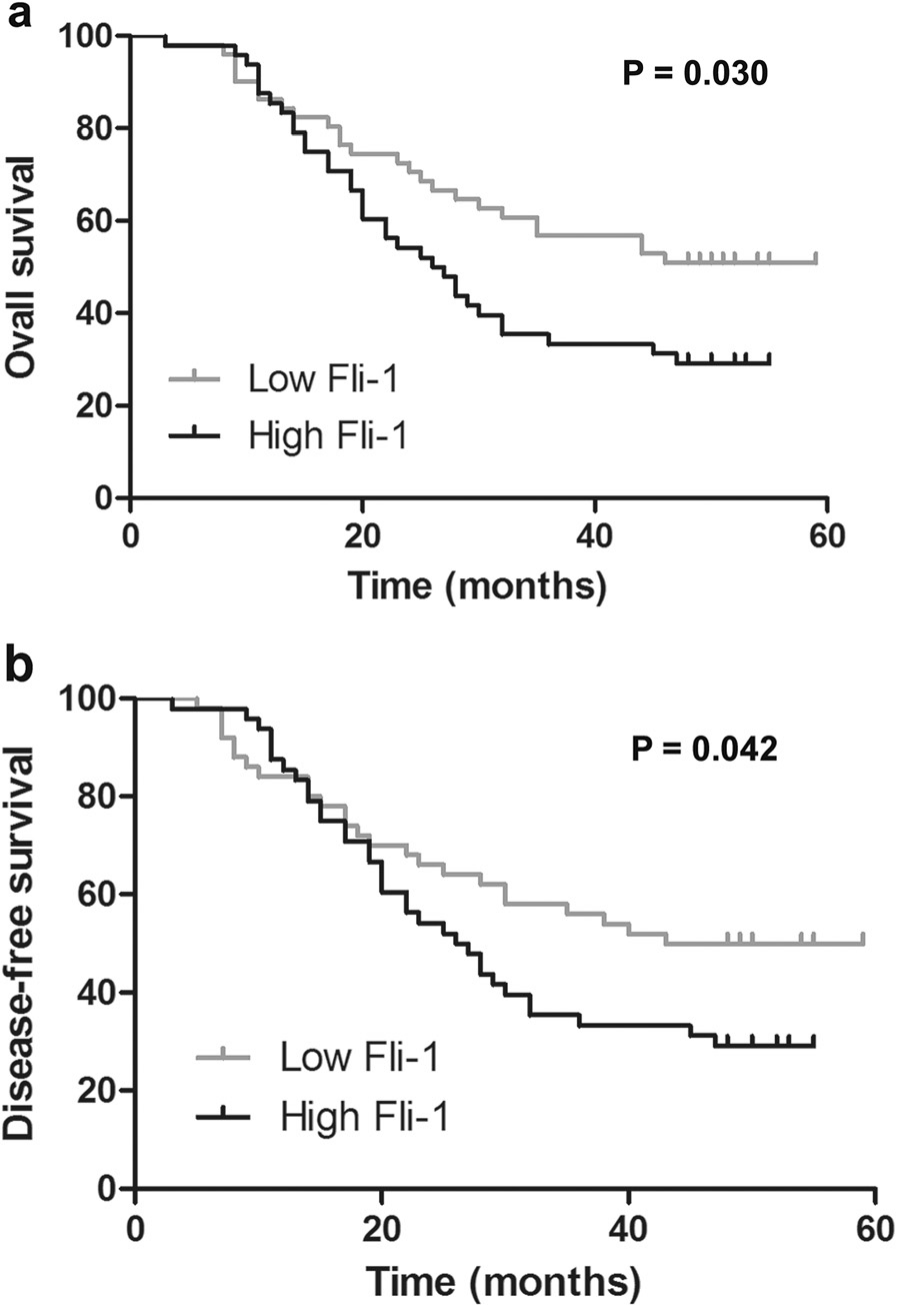
**Fli-1 predicts clinical outcome of ovarian cancer. (a, b)** Kaplan-Meier estimates of overall survival **(a)** and disease-free survival **(b)** in 99 EOC patients. *P* value refers to two-sided log-rank tests.

The median follow-up interval was 32.8 months. Five patients were lost during follow-up. In univariate survival analyses for OS and DFS, 99 EOC patients were divided into two groups based on Fli-1 expression score in tumors, representing low (scores 0–1.1) and high (scores > 1.1) expression of Fli-1. The Kaplan Meier survival curve in Figure 3 confirmed that patients with low expression of Fli-1 had better OS (*p* = 0.030) and DFS (*p* = 0.042). The median OS for the high Fli-1 expression group (48 patients, 33 events) was 27 month; however, the low Fli-1 expression group had significantly longer survival (48 months) (51 patients, 25 events). The median DFS was 23 month for the high Fli-1 expression group (48 patients, 33 events) but 43 month for the low Fli-1 expression group (51 patients, 26 events).

### Knockdown of Fli-1 inhibits cell proliferation in SKOV3 cells

**Figure 4:**
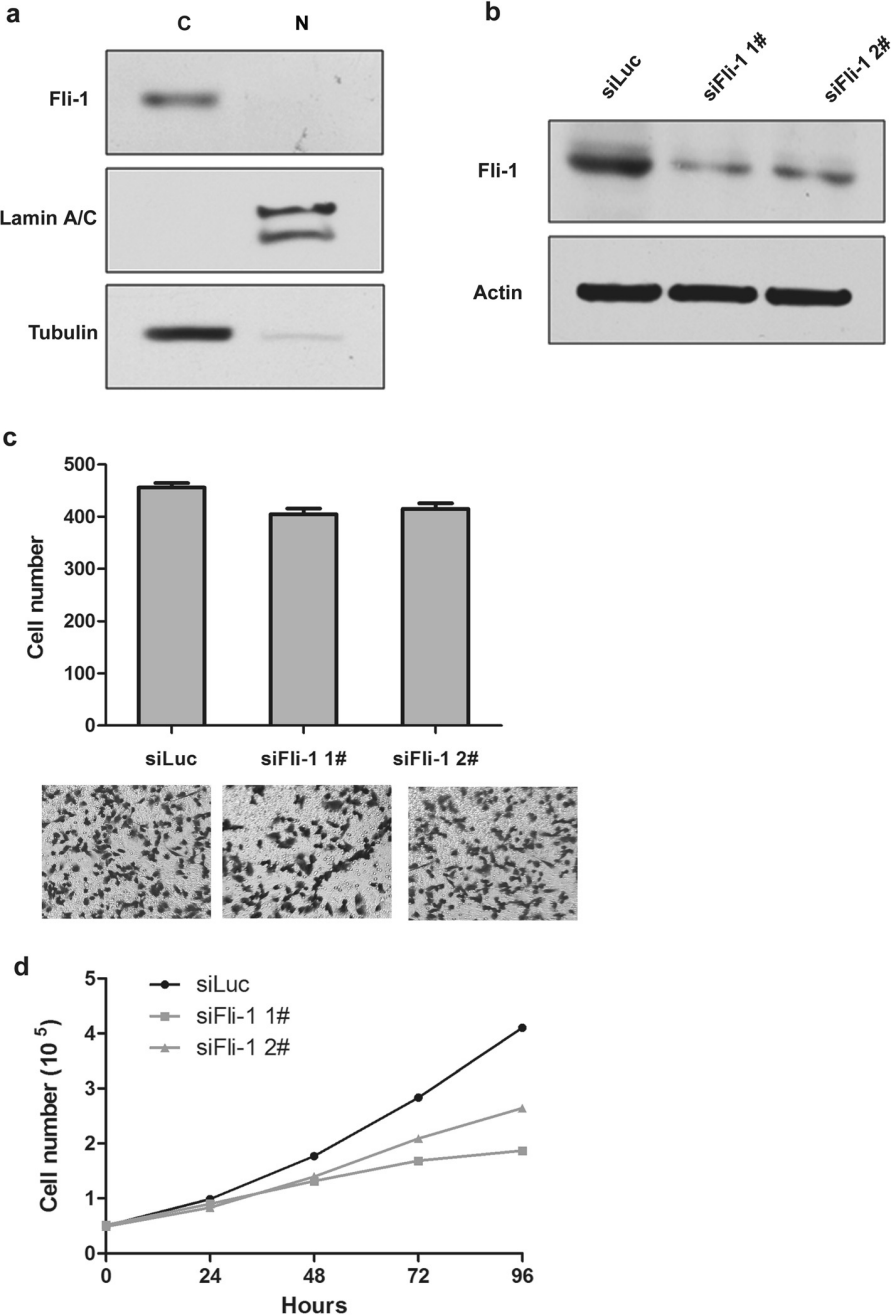
**Present of Fli-1 and growth characteristics of SKOV3 cells with Fli-1 expression down-regulated. (a)** Western blots showing the purity of the isolated nucleus/cytoplasm sample, nuclear (N) and cytoplasmic (C). **(b)** SiRNA transfection efficiency in tumor cells was measured by Western blotting; **(c)** Transwell migration assay of the indicated cell lines transfected with Fli-1 constructs or transient transfected with two different Fli-1 siRNA target sequence. **(d)** The growth curve displays the absolute counts of cells cultured in twelve-well plates during the 4-day treatment.

The cellular localization of Fli-1 was further examined in SKOV3 cells. The fractionation was verified by the presence of Lamin A/C in nuclei and tubulin in cytoplasm, and Fli-1 was present in the cytoplasm (Figure 4a).

Fli-1 was knocked down with target siRNA sequences in SKOV3 cells and the efficiency was detected by Western blotting (Figure 4b). Initial microscopic observation and cell counting with Trypan blue showed that the proliferation of the cells treated with Fli-1 siRNA was significantly reduced (Figure 4d, *p*< 0.01). The capability of migration of SKOV3 cells treated with control siRNA or Fli-1 siRNA were also examined. As shown in Figure 4c, knocking-down Fli-1 expression, however, did not have impact on the migration capacity (*p*> 0.05).

## Discussion

EOC is a very aggressive gynecological tumor. Despite the use of multimodal therapy, their prognosis remains poor, with the probability of 5 years survival less than 30% for those presenting with advanced disease [24-26]. The molecular mechanisms involved in EOC remain largely unknown, and neither was the prediction biomarker for prognosis.

The present study is dedicated to identify biomarkers for prediction and intervention in the tumorigenesis and development of EOC. To study the association between Fli-1 and EOC, the expression of Fli-1 in EOC was detected by immunohistochemistry. Approximately 90% of ES/PNET had a specific t(11; 22)(q24;q12) that results in fusion of the EWS and FLI-1 genes, and overexpression of FLI-1 protein. Therefore, PNET was used as positive control. The expression of Fli-1 in PNET was located in the nucleus. In contrast, we found that Fli-1 was predominantly located in the cytoplasm in 74% cases with various intensities. In recent years, with the full realization of the genesis for ovarian cancer, it is strongly suggested that high grade ovarian cancer originates not from the surface of the ovary, but from the epithelial layer of the neighboring fallopian tube epithelium [27,28]. Therefore, fallopian tube tissues were taken for control group together with normal ovaries.

The Fli-1 expression was negative in control group, but increased in early-stage tumors, and reached the highest level in advanced stage tumors. Clinicopathologic analysis of Fli-1 expression revealed that the high expression of Fli-1 was positively correlated with advanced tumor stage and positive lymph nodal involvement. This progressively increased expression profile paralleled with deterioration of the disease, suggested a role of Fli-1 in progression of EOC. Although it was shown no significant association between Fli-1 expression and histological grade, the imbalance in sample size between low grade (G1, 10) and high grade (G2 and G3, 94) should be considered. At the same time, the study showed that high expression of biomarker CA125 was related to the staining of Fli-1, and the significance needed to be investigated.

The relationship between Fli-1 expression and prognosis was further analyzed by OS and DFS. Patients with high expression of Fli-1 had poor OS and DFS, suggesting that Fli-1 is an attractive candidate for risk prognostication and the target therapy of EOC. As the treatment would have impact on survival, we also analyzed the treatment in the two groups. In this study, all of the patients were treated with standard regimens. Therefore, Fli-1 expression is highly associated with the survival in the patients with ovary cancer.

Increasing expression of Fli-1 is one of the common scenarios during tumor development and may be associated with the disease malignancy. To further study the role of Fli-1 overexpression in growth and metastasis, the function of Fli-1 in cell line was investigated. Functionally, we found knocking-down of Fli-1 reduced ovarian cancer cell proliferation, but did not affect tumor metastasis.

The expression of Fli-1 was predominantly found in the nuclei of Ewing sarcoma and leukemia [29]. In the present study, Fli-1 expression was mainly found in the cytoplasm of ovarian cancer tissues and SKOV3 cells. These data suggest that Fli-1 is required to function in the cytoplasm for ovarian cancer. Moreover, previous studies support that Fli-1 might function through protein-protein interaction or as being a transcription factor [15,16,30,31]. It was speculated that Fli-1 were widely expressed in various cancer tissues while it specifically played different roles. Thus, our results imply that Fli-1 may have distinct functions in signal transduction pathways in the cytoplasma, other than just being transcription factor. The status of Fli-1 in different cancers and the clinical implications of their expression during cancer development still need further investigation. In addition to the functional study, further investigation of the molecular mechanisms of Fli-1 is warranted.

Although invasive epithelial ovarian cancer is widely seen and treated as a single disease entity, there are different histological subtypes. Serous ovarian cancer studied in this study is the most common subtype. The expression status of Fli-1 in other subtypes also needs to be investigated in the future.

More importantly, we demonstrate a significant correlation between high Fli-1 immunoreactivity and shorter overall and disease-free survival. If high Fli-1 expression can be further confirmed to indicate poor prognosis, as suggested in this report, it may serve as an important prognostic marker and an attractive therapeutic target in ovarian cancer. However, this study has limitation in sample size, and it is a retrospective and monocentric study. Therefore, further larger, multicentric studies are needed.

## Conclusion

In conclusion, our findings suggest that Fli-1 is an important molecular change significantly related to tumorigenesis and progression of EOC. However, a larger cohort of patients with ovarian cancer and other cancer types is still required to further define the clinical significance of Fli-1 and its prognostic value in ovarian cancers in the future.

## Abbreviations

EOC: Epithelial ovarian cancer; Fli-1: Friend leukemia virus integration 1; IHC: Immunohistochemical; FIGO: Federation of Gynecology and Obstetrics; OR: Odd ratio; DFS: Disease free survival; OS: Overall survival.

## Competing interests

The authors declared that they have no financial or non-financial competing interests.

## Authors’ contributions

WS and LYH designed experiments, carried out the laboratory experiments, analyzed the data, interpreted the results and wrote the paper. WL and GJW participated in the design of the study and discussed analyses. YL and LY performed cell culture and transfection. ALL and JWC contributed the conception and design of this study, and helped to draft the manuscript. All authors read and approved the final manuscript.

## Pre-publication history

The pre-publication history for this paper can be accessed here:

http://www.biomedcentral.com/1471-2407/14/424/prepub
